# The Children's Hospital at Nottingham

**Published:** 1899-01-21

**Authors:** 


### THE CHILDREN'S HOSPITAL AT NOTTINGHAM.

In publishing the following correspondence, we would point out that we make no charge and bring no complaint against the Children's Hospital at Nottingham. But we are strongly convinced of the desirability of all charges brought against hospitals being publicly met and openly investigated, and the charge here brought against the management of the Nottingham Children's Hospital is far too serious to be passed over without full explanation. At first sight it may be thought by some who read the correspondence that a full denial has been given to the allegations made by Miss Morrish; but we do not feel quite satisfied that this is so, and we think that the matter should be cleared up. In saying this we do not concern ourselves so much with the discomforts to which the nurse is stated to have been exposed, as with the system of leaving responsible work to untrained probationers. It will be observed that Miss Morrish says: "I had only been at the hospital about three weeks when I was placed upon 'isolation duty.' . . . If I could catch any sleep I was compelled to take it in the ward with the patient. . . . The only mode of communication with the rest of the hospital staff or otherwise—day or night—was by opening a window and ringing a small hand-bell. The patient I had charge of was a case of traumatic erysipelas of the head and face which, later on, extended to the chest, arms, and abdomen. For days it was totally blind. . . . That the case was a most critical one is evidenced by the fact that the clergyman was called in to baptise it. Although I had never done such things before I had to give it enemas. . . . I had only one hour off duty in the twenty-four. . . . Whilst on 'isolation duty' I was never once visited during the night. On more than one occasion I was not visited—except by the servant that placed my food on the stairs—from early in the afternoon until about noon the next day." Now we need hardly say that the gravity of these allegations lies in the fact that an untrained woman was put to do work which should only have been undertaken by one who had obtained a very considerable practical experience in the nursing of children, and we cannot but feel that it is no refutation of the charge to say as is done in the Answer No. 1, given below, that "Miss Morrish was only a 'sitter,' the sister in charge being in charge of and visiting the case at her discretion"; or, as in Answer No. 4, that "visits at night are made to the isolation ward at the discretion of the sister in charge." The point is, How far did her discretion carry her? Can it be said that the sister in charge was really in charge of the child if Miss Morrish is correct in stating that she was "never once visited during the night," and that on more than one occasion she "was not visited from early in the afternoon until about noon the next day." It is idle to say that the isolation ward is visited at night "at the discretion of the sister in charge" if the discretion of this lady leads her not to visit it at night at all. We think the whole affair demands a complete investigation and a much more full explanation than has been given us. The very carefulness of the wording of the "Answers" makes us question whether we have got at the bottom of the matter. We asked whether a probationer of only three weeks' standing has been placed "in charge" of a patient, and we are told that she has not been placed "in sole charge." Then we do not like the use made of the term "sitter." When a woman is left alone with a sick child for upwards of twelve hours at a stretch, and when that child is suffering from erysipelas which is sufficiently extensive to implicate the head, chest, arms, and abdomen, and sufficiently severe to cause swelling of the lips and closure of the eyes, and to lead to the child being baptised, and when, among other duties, she has to administer enemas and take temperatures, surely such a woman is doing nurse's work and cannot be fairly called a "sitter." Under these circumstances, we think it best at once to publish the whole correspondence, for we cannot but feel that either the allegations made ought to be much more fully refuted than has been done, or that the abuses indicated should be reformed. One or the other.

"To the Editor of THE HOSPITAL.

"SIR,—Attracted by an advertisement appearing in THE HOSPITAL, my daughter, Catherine Ada Morrish, applied for the position of Probationer Nurse at the Free Hospital for Sick Children, Nottingham, and receiving an appointment, subject to a month's trial, entered upon her duties on August 29th. The treatment she received will, I think, be best understood from the accompanying detailed statement, which I beg to send for publication, in the hopes of saving other inexperienced girls from like treatment, and other poor helpless patients from such so-called professional nursing. I feel that I should be wanting in my duty to the public were I not to bring this matter to your notice.—Yours faithfully,

"T. FOX MORRISH, M.R.C.S. Eng.

"4, Dingle Hill, Prince's Park, Liverpool,
"December 12th, 1898."

---

STATEMENT BY CATHERINE ADA MORRISH, late a Probationer Nurse at the Nottingham Free Hospital for Children.

"I had only been at the hospital about three weeks when I was placed upon 'Isolation duty.' Up to this time I had been taught very little, my hospital work being principally manual. The following is a true account of my experience on 'Isolation duty': The building is away from the rest of the hospital. It is approached from outside by a stone staircase which gives access to a passage, at one end of which is a ward. This contains a bed for the patient, a bed for the nurse, a table upon which are placed various articles for the doctor, several chairs, one common wooden rocking chair without arms, a washstand with a jug and basin, which was called the doctor's basin, his towel being placed through the handle of the jug. A small room opens directly out of this ward. It contains a spare bedstead, upon which my bed was rolled during the day, and a dressing-table or washstand—it might be either—without basin, &c. At the other end of the passage is a closet that contains a sink directly under the window; the w.c. opens out of this closet. If I could catch any sleep I was compelled to take it in the ward with the patient. I had also to have my food there, or in the small room adjoining. If I did the latter I had to eat off the washstand, being on duty all the time. My food was placed at the bottom of the stone steps outside, and I went down and fetched it. Any ablutions I performed had to be accomplished in the doctor's basin or in the sink. The only soap supplied to me—with the exception of a small cake for the doctor, and which was marked "Visitors"—was a piece of Sunlight soap, with which I also washed the baby. One of the nurses said to me, 'If you want to wash in the sink be sure to put something over the window, there is no blind.' The only mode of communication with the rest of the hospital staff or otherwise, day or night, was by opening a window and ringing a small hand bell. The patient I had charge of was a case of traumatic erysipelas of the head and face, which later on extended to the chest, arms, and abdomen. For days it was totally blind. It was a child twelve months of age, and up to the time of admission had been on the mother's breast. It also suffered from painful dentition. That the case was a most critical one is evidenced by the fact that the clergyman was called in to baptize it. Although I had never done such things before I had to give it enemas, and also to take the temperature; which I believe was very high, as the case was said to be severe. I had to give the child medicine every three hours. It was fed on milk and water from the medicine glass, no other vessel being supplied. This was most difficult, as the child had not been weaned, and its lips were so swollen. I had only one hour off duty in the twenty-four. On Sunday I was off duty for one and a half hours or two hours for church. Whilst on 'isolation duty' I was never once visited during the night. On more than one occasion I was not visited—except by the servant that placed my food on the stairs—from early in the afternoon until about noon the next day. I was kept on this duty for twelve consecutive days and nights, when my father sent for me to come away.

"C. A. MORRISH."

On receiving the above we put ourselves in communication with the secretary of the Nottingham Children's Hospital, from whom we received a letter, marked "private and confidential," asking us, in case we should decide to publish what Mr. Morrish had sent to us, to publish at the same time the following communication which he had sent to Mr. Morrish:—

[COPY.]

"Free Hospital for Sick Children, Nottingham,
"28th October, 1898.

"T. F. Morrish, Esq., M.D., Liverpool.

"Dear Sir,—Your letter and the statements of yourself and your daughter (dated 13th October and enclosed therein) have received the careful attention of the board of this hospital, and their contents have been closely investigated. We believe that many of the points have already been dealt with in a letter from the lady superintendent to yourself, and therefore we do not propose to reply categorically now. As the general result we are convinced that you are entirely under a misapprehension in attributing to anyone connected with this hospital anything in the nature of 'cruelty' or any intention to 'impose upon' or to 'drive' your daughter from the institution; nor can we admit various other of the allegations against the hospital and its staff. If such allegations were well founded your daughter would certainly not have volunteered the statement (shortly before she left) that she had been happy during her stay here; and the board would hardly be able to look back over the past twenty years without finding some similar complaint either from a nurse or her friends. No such complaint, however, has reached us until we received yours; on the contrary, during that period a large number of nurses have passed through the hospital (including isolation) and expressed every satisfaction with their life here. Your daughter's statement contains, we understand, several inaccuracies, introduced, perhaps by inadvertence or misapprehension of the instructions given to her, and we cannot but think that the hardships which she specifies were due, at any rate, partially to her disregard of the arrangements at her disposal. We are assured that she was daily asked whether she had all that she wanted, and everything that she mentioned was at once provided.

"Having said so much, we think it right to add that we cannot regard the structural arrangements of this hospital (and especially the isolation block) as ideal, nor can we feel that the nursing staff is at present (partly owing to recent illnesses) as ample as we should like to see it. Much improvement has been effected in recent years, and we are constantly endeavouring to introduce larger reforms, but you, as a medical man, must know the difficulty as to ways and means that so generally hampers the governing bodies of such institutions. Meantime we have to make the best of the machinery at our command, with what success the past record of the hospital shows. But your letter has brought before us emphatically how very difficult it is for the regulations of the hospital to be carried out strictly in the present isolation block, particularly in the case of an inexperienced nurse; and the board are considering how it is possible to remedy this state of things. This result will no doubt give you satisfaction.—I am, sir, yours obediently,

"H. C. RUSSELL,
"Chairman of the Board of Management."

"22nd December, 1898.

"Dear Sir,—In reference to your reply to our communication we beg to remark that the letter which was sent to T. F. Morrish, Esq., is in no wise an answer to the questions which we asked you. We are not so much concerned as to whether Miss Morrish was comfortable at the Children's Hospital or not; but it is in our opinion an exceedingly grave matter as to whether your hospital authorities permitted a probationer of three weeks' standing to take sole charge of a case in the isolation ward. We shall be very glad to receive a definite denial of this point from you before considering the matter further ourselves.—We are, yours faithfully, "(Signed) THE EDITOR.

"The Secretary, Free Hospital for Sick Children, Nottingham."

In answer to this we received the following:

CHILDREN'S HOSPITAL, NOTTINGHAM.

Answers to questions asked by the Editor of THE HOSPITAL respecting a complaint against the management of the above institution.

| *Question.* | *Answer.* |
|---|---|
| 1. That on any occasion a probationer of only three weeks' standing has been placed in charge, day and night, of a patient in the isolation ward? | It is not true that a probationer of three weeks' standing has been placed in sole charge of a case day and night. Miss Morrish acted only as "sitter," the sister in charge being in charge of, and visiting the case at her discretion, who also undertook the dressing and attended to all the medical details connected with the case. |
| 2. Is it also a fact that no place is provided for the nurse having charge of a case in the isolation ward to procure her meals in comfort? That is to say, is there no table provided in the bedroom for the nurse's meals? | Tables have always been provided, both in the ward and nurse's bedroom. |
| 3. Also, is it a fact that any nurse has been asked to undertake the charge of a case without a basin being provided in her bedroom for her ablutions, the basin being for her sole use? | The nurse's basin has certainly been used by the medical officer, but this is at the option of the nurse herself, who can obtain another if she should prefer it. Usually the nurse sees no objection to the use of her basin. |
| 4. Is it a fact that the nurse on duty in the isolation ward receives no visit from anyone during the night? | Visits at night are made to the isolation ward at the discretion of the sister in charge. The isolation ward is within a few feet of the main building. It may also assist the Editor to know that the particular case under treatment was an unimportant one. |

H. C. RUSSELL, Chairman of Board.
LEWIS W. MARSHALL, } Medical
A. W. CHALMERS PESKETT, } Officers.
LILIAN PARMITER, Lady Superintendent.

